# Diversity, ecology and distribution of benthic diatoms in thermo-mineral springs in Auvergne (France) and Sardinia (Italy)

**DOI:** 10.7717/peerj.7238

**Published:** 2019-07-15

**Authors:** Giuseppina G. Lai, Aude Beauger, Carlos E. Wetzel, Bachisio M. Padedda, Olivier Voldoire, Antonella Lugliè, Elisabeth Allain, Luc Ector

**Affiliations:** 1 Department of Architecture, Design and Urban Planning, University of Sassari, Sassari, Italy; 2 CNRS, GEOLAB, University Clermont Auvergne, Clermont-Ferrand, France; 3 Environmental Research and Innovation Department (ERIN), Luxembourg Institute of Science and Technology (LIST), Belvaux, Luxembourg

**Keywords:** Springs, Biodiversity, Thermo-mineral waters, Bacillariophyceae, Auvergne, Sardinia, Extreme conditions

## Abstract

This study investigated and compared the diatom flora from thermo-mineral springs in Auvergne (France) and Sardinia (Italy). Samples were collected from rock/cobbles and fine sediments in 16 springs between January 2015 and March 2017. A total of 207 taxa (59 genera) were found. Multivariate analyses revealed significant differences in species composition and abundance among diatom assemblages both within each geographic region and between Auvergne and Sardinia (global *R* = 0.516; *p* = 0.002), suggesting the importance of local and climatic factors in species distribution. Based on abundance and common occurrence in multiple sites, some taxa can be considered more representative of springs in each region: *Crenotia thermalis* for Auvergne and *Lemnicola exigua*, *Nitzschia amphibia*, *N. inconspicua* and *Rhopalodia operculata* for Sardinia. pH, conductivity and HCO_3_^−^ were the most significant environmental variables for diatom assemblages. Our results highlight the high heterogeneity of these spring systems. Future taxonomic insights can be useful to define the identity of some abundant and dominant taxa not identified at the species level in this study. Their identification is a crucial step for a more precise ecological characterization and comparison of these peculiar spring systems.

## Introduction

Thermo-mineral springs are a diverse group of aquatic environments with characteristics that make them very good prospects for different uses including industrial processing, bottled water, power generation, and in the health and well-being sector. The interest in this resource, used since ancient times, has greatly increased over the past few decades (Durowoju, Odiyo & Ekosse, 2018). The knowledge of these springs’ biotic communities, traditionally neglected when compared to physical and chemical ones, is a prerequisite for their proper use and management. In fact, the abundance and diversity of spring-dwelling organisms, besides having intrinsic value, provide a number of ecological goods and services (Boothroyd, Hay & Turner, 2006). Thermo-mineral springs are considered very interesting habitats for the study of algal microflora, including diatoms, which are often abundant and able to survive in different ecological niches (Nikulina & Kociolek, 2011; Leira, Meijide-Failde & Torres, 2017). They can host distinctive assemblages of species, some of them well adapted to extreme and hostile conditions (Pumas, Pruetiworanan & Peerapornpisal, 2018). Further, diatoms are very sensitive to a variety of environmental conditions and can provide important information on the ecological integrity of these ecosystems (Niyatbekov & Barinova, 2018). In Europe, investigations on diatom flora in thermo-mineral springs have been carried out in the Czech Republic (Bílý, 1934; Lederer et al., 1998; Kaštovský & Komárek, 2001), France (Beauger et al., 2015; Beauger et al., 2016; Beauger et al., 2017), Iceland (Schwabe, 1933; Schwabe, 1936; Krasske, 1938; Van der Werff, 1941; Biebl & Kusel-Fetzmann, 1966; Owen, Renaut & Jones, 2008), Italy (Dell’Uomo, 1986; Mannino, 2007; Lai et al., 2019), the Republic of Macedonia (Stavreva-Veselinovska & Todorovska, 2010), Slovakia (Hindák & Hindáková, 2006; Hindák & Hindáková, 2007), and Spain (Leira, Meijide-Failde & Torres, 2017). Overall, these studies were limited and focused on individual or multiple springs in specific geological settings and the comparison between different geographic areas is rare (e.g., Petersen, 1946; Owen, Renaut & Jones, 2008).

This study compares the diatom assemblages from the thermo-mineral springs of Auvergne (Massif Central, France) and different areas of Sardinia (Italy). A crustal continuity between Sardinia and southwestern France (Provence) up to the Oligo-Miocene was hypothesized by several authors (e.g., Carmignani et al., 1994; Cassinis, Durand & Ronchi, 2003; Elter et al., 2004). In addition, paleontological affinities, particularly micro and macrofloras, seem to confirm close relationships between Sardinia and the southern border of France, including inner sectors of the Massif Central (Ronchi et al., 1998; Broutin et al., 2000). We hypothesized significant differences among diatom assemblages between Auvergne and Sardinia, despite their possible common geological history, and among diatom assemblages within each geographic region since both local and climate-related factors are considered important drivers for growth and distribution diatom species (e.g., Patrick, 1948). The main objectives of this study were: (1) to describe the species composition and structure of diatom assemblages; (2) to examine the degree of similarity/dissimilarity among diatom assemblages within each geographic region and between Auvergne and Sardinia; (3) to explore the relationships between diatom taxa and environmental variables.

## Materials & Methods

### Study sites

**Figure 1:**
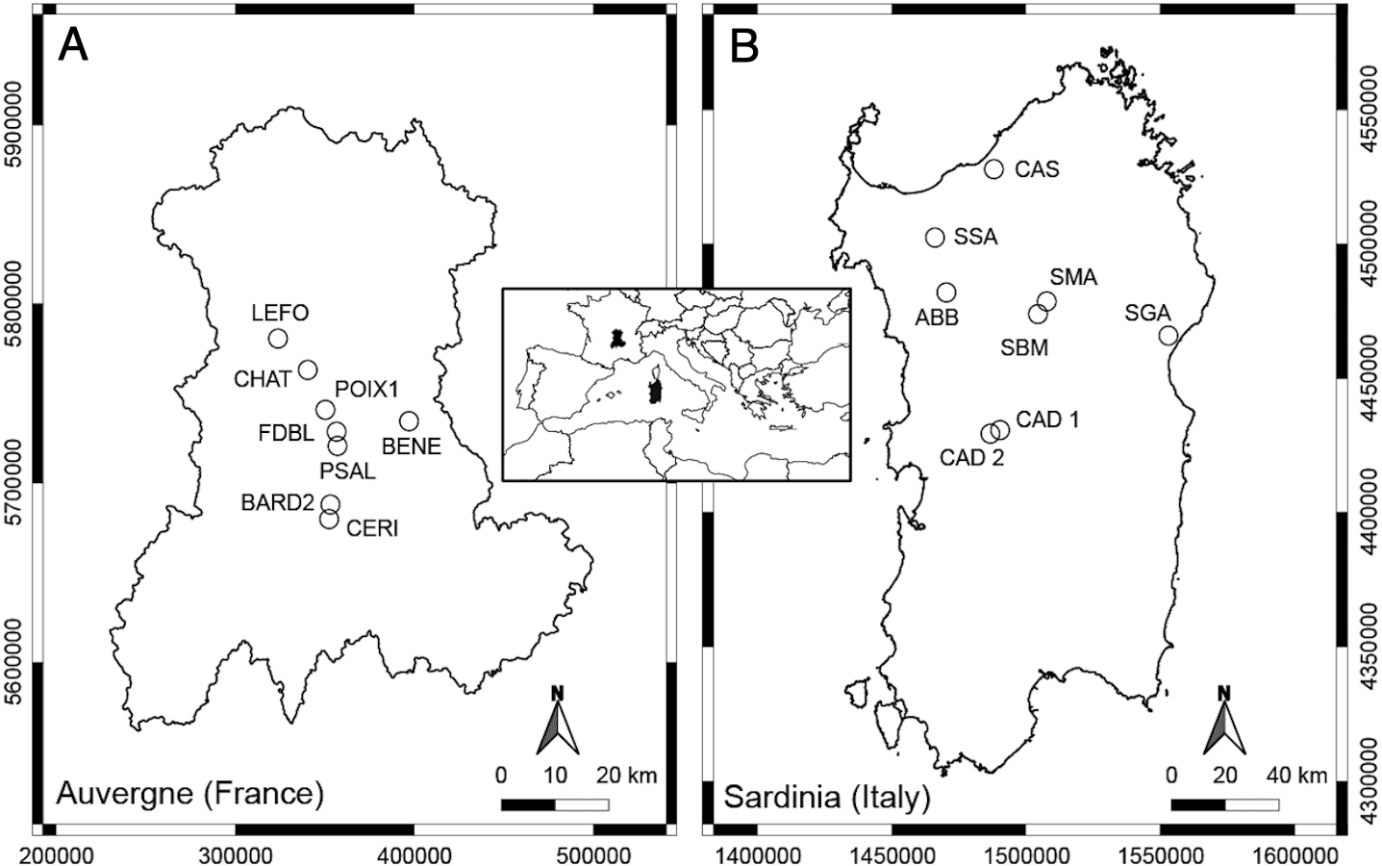
Geographic position of the studied springs in Auvergne, France (A) and Sardinia, Italy (B).

The thermo-mineral springs studied in Auvergne (French Massif Central) are mainly located in the department of Puy-de-Dôme, as the majority of springs are located in this area (Fig. 1, Table 1). The Massif Central is an old granitic shield formed during the Hercynian orogenesis. With Alpine and Pyrenean orogenesis, dislocation of the shield appeared, and volcanic formations occurred in the Tertiary and Quaternary periods (Mottet, 1999). The bedrock of the Massif is mainly granite and gneiss. At Châteauneuf-les-Bains and Les Martres-de-Veyre, springs LEFO, FDBL and PSAL come directly from granite. Moreover, FDBL and PSAL are located just above two principal fault systems, which are certainly inherited from the tardi-Hercynian period (Battani et al., 2010). At Châtel-Guyon, Courpière and Augnat, springs CHAT, BENE and CERI lie along a fault line. At Boudes, spring BARD2 emerges from the fractured Hercynian metamorphic basement (Négrel et al., 2000). The spring POIX1, at Clermont-Ferrand, emerges using a former volcano chimney crossing a layer of bitumen. Most of these springs have a high content of free CO_2_, and CHAT, PSAL and POIX1 have high sodium bicarbonate content (Boineau & Maisonneuve, 1972). The majority of the selected springs, known since the Gallo-Roman period, are rheocrenic systems not used by local populations.

**Table 1:** Main characteristics and use of the studied springs.

Spring	Abbreviation	Municipality	Altitude m a.s.l	Type	Captation	Use
**Auvergne-France**						
Petit Saladis	PSAL	Les Martres-de-Veyre	345	R		nu
Font de Bleix	FDBL	Les Martres-de-Veyre	332	R		nu
Bard 2	BARD2	Boudes	496	R		nu
Lefort	LEFO	Châteauneuf-les-Bains	379	R-M	X	nu
Cerisier	CERI	Augnat	497	R		nu
Châtel-Guyon	CHAT	Châtel-Guyon	421	R-M	X	t
Bénédictins	BENE	Courpière	331	R-M	X	nu
Poix 1	POIX1	Clermont-Ferrand	337	R		nu
**Sardinia -Italy**						
S. Giovanni Su Anzu	SGA	Dorgali	151	R-M	X	ftb
Caddas 1s	CAD1	Fordongianus	22	R		ftb
Caddas 1d	CAD2	Fordongianus	22	R		ftb
San Saturnino	SSA	Benetutti	252	R-M	X	ftb
Abbarghente	ABB	Romana	145	F	X	d
Su Banzu Mannu	SBM	Orani	205	R-M	X	ftb
San Martino	SMA	Codrongianos	211	L		nu
Casteldoria	CAS	Santa Maria Coghinas	7	R-M	X	t

**Notes.**

Type R rheocrenic R-M rheocrenic modified L limnocrenic

Use nu not used t therapeutic ftb free thermal baths d drinking

The thermo-mineral springs selected for Sardinia are located in the main geothermal areas of the central-northern part of the Island (Fig. 1; Table 1). It is not clear if these aquatic systems are linked to recent volcanic activity, but they are mostly located along a complex crustal fault system generated by the Cenozoic geodynamic (Cuccuru, Oggiano & Funedda, 2015). This fault system forms a plumbing network for water coming from deep-seated reservoirs heated by the relatively high thermal gradient (Cuccuru, Oggiano & Funedda, 2015). At Fordongianus and Romana, springs CAD1, CAD2 and ABB are located on alluvial sediments. At Casteldoria, Benetutti and Oddini, springs CAS, SSA and SBM emerge from granites. At Codrongianos and Dorgali, springs SMA and SGA are respectively located on ignimbrites and carbonate sediments. These springs are mostly captured rheocrenic systems and have different water chemistry (e.g., Dettori, Zanzari & Zuddas, 1982). Gaseous emanations with a prevalence of CO_2_ are present at springs ABB and SMA, and those with a prevalence of N_2_ at CAS, CAD1, CAD2 and SBM (Dettori, Zanzari & Zuddas, 1982). Spring SMA is the only limnocrenic spring and differs from the others by the presence of significant incrustations of travertine and iron oxides (Dettori, Zanzari & Zuddas, 1982). Most of these springs, known since Roman times, are currently used as free thermal baths.

### Sampling

Diatom samples and water samples for physical and chemical analyses were simultaneously collected in winter in eight springs in Auvergne and in eight springs in Sardinia. In Auvergne, eight composite diatom samples from epilithon and epipelon were collected once, between January 2015 and March 2017. In Sardinia, 16 diatom samples were collected once, separately from epilithon and epipelon (two samples in each site), between January and February 2017.

Diatoms were collected on rock/cobbles (depending on the availability of substrate) and fine sediments using a hard-bristled toothbrush and glass tubes in the entire spring area, following the methods reported in Kelly et al. (1998) and ISPRA (2014). All diatom samples were preserved in 100 mL polyethylene bottles and fixed *in situ* with a formaldehyde solution (4% v/v).

Water samples were collected using 1-L polyethylene bottles and were analysed in the laboratory within 24 h from sampling. In the springs of Sardinia, water samples were also collected in 150 mL glass bottles and immediately fixed *in situ* for the analysis of dissolved oxygen in the laboratory, following the Winkler method (Winkler, 1888).

### Measurements and analyses

In Auvergne, the geographic position of the sites was recorded using a DGPS Trimble Geo7x. The water temperature, pH and conductivity were measured *in situ* with a WTW Multiline P4, and the dissolved oxygen with an oximeter Ysi ProODO. The water samples were analysed in the laboratory using the high-pressure ion chromatography technique following filtration with Whatmann GF/C filters. The Thermo Scientific Dionex ICS1100 and Thermo Scientific Dionex DX120 systems were used for the analyses of cations and anions, respectively.

In Sardinia, the geographic position of the springs was recorded using a GPS Garmin eTrex Vista HCx. The water temperature was measured *in situ* using a digital thermometer (Temp 7 RTD basic with immersion probe PT100) in the warmer springs (CAD1, CAD2 and CAS). In the cooler springs (SGA, SSA, ABB, SBM, and SMA) water temperature, pH and conductivity were measured using a multiparameter probe (YSI ProPlus). The water samples were analysed in the laboratory following standard methods reported by APAT & IRSA-CNR (2003), APHA (1998) and Strickland & Parsons (1972). Dissolved oxygen was analysed using the Winkler method (Winkler, 1888).

Overall, the hydrochemical variables analysed were: sodium (Na^+^), potassium (K^+^), magnesium (Mg^2+^), calcium (Ca^2+^), bicarbonate (HCO_3_^−^), fluorine (F^−^), chlorides (Cl^−^), bromine (Br^−^), sulfate (SO4^2−^), ammonia nitrogen (N-NH_4_^+^), nitrites (N-NO_2_^−^), and nitrates (N-NO_3_^−^). Total nitrogen, reactive phosphorus and total phosphorus were analysed only for springs in Sardinia.

A part of the diatom samples (50 mL) were treated by an oxidation process on a heating plate with H_2_O_2_ (30% v/v), and, if necessary, HCl (37% v/v) was added to remove carbonates. Cleaned diatoms were mounted on microscope slides using Styrax (refractive index = 1.59) and Naphrax (refractive index = 1.73) resins. Diatom observations and counts were performed using light microscopy (LM, Zeiss Axiovert 10 and Leica DM2700M microscopes equipped with phase-contrast and micrometric scale) at 1000 × magnification. Cleaned diatoms from the same treated samples were also mounted on aluminium stubs, coated with platinum, and analysed by a scanning electron microscope (SEM, Hitachi SU-70).

Diatom species were identified according to Krammer & Lange-Bertalot (1986), Krammer & Lange-Bertalot (1988), Krammer & Lange-Bertalot (1991a), Krammer & Lange-Bertalot (1991b), Krammer (2000), Krammer & Lange-Bertalot (2000), Lange-Bertalot (2001), Lange-Bertalot et al. (2003), Werum & Lange-Bertalot (2004), Levkov (2009), Żelazna Wieczorek (2011), and recent literature (e.g., Beauger et al., 2015; Wetzel et al., 2015; Beauger et al., 2016; Beauger et al., 2018).

### Data processing and statistical analyses

Diatom count data from the epilithon and epipelon samples, analysed separately for springs in Sardinia, were integrated in a single data set and then mediated to have homogeneous data comparable with those from Auvergne.

The structure of diatom assemblages was examined by species richness (SR), the Shannon-Wiener diversity index (H′) (Shannon & Weaver, 1949) and Pielou’s evenness index (J′) (Pielou, 1975), calculated using OMNIDIA 6.0 software (Lecointe, Coste & Prygiel, 1993). The ecological preferences of taxa with respect to pH reaction, salinity, trophic state and moisture were attributed by consulting Van Dam, Mertens & Sinkeldam (1994).

All diatom count data were converted in relative abundances (RA) for the statistical analyses.

The similarity among assemblages was analysed using non-metric multidimensional scaling ordination (nMDS). A Bray–Curtis similarity matrix was constructed using log (x + 1) abundance data from all species with a relative abundance >1%. The significance of the differences was validated by a one-way analysis of similarities (ANOSIM). For this analysis, probability (*p*) < 0.03 was considered significant. To support ANOSIM, the percentage level of similarity of diatom assemblages and the percentage contribution of each species to the differences were determined using SIMPER analysis. The species were classified from highest to the lowest contribution to identify the sub-group whose cumulative percentage contribution reached 70% of the dissimilarity value. nMDS, SIMPER and ANOSIM were performed using PRIMER 5 (Clarke & Gorley, 2001). Relationships among environmental variables and patterns of sampling sites were analysed using a Principal Component Analysis (PCA) based on a data matrix built with environmental variables log (x + 1) transformed (except for pH). PCA analysis was performed using the software Canoco 4.5 (Ter Braak & Šmilauer, 2002).

Relationships between diatom species and environmental variables were explored by a Canonical Correspondence Analysis (CCA) using Canoco 4.5 (Ter Braak & Šmilauer, 2002), after the previous assessment of the length of gradient (>4) by means of a Detrended Correspondence Analysis (DCA) of diatom data. Physical and chemical data (except for pH), and diatom data, were log (x + 1) transformed. All canonical axes were used to assess the significant variables through analyses by means of a Monte Carlo test (1000 permutations). The ordination plot was split into two different plots for sites and diatom species.

**Table 2:** Results of the physical and chemical variables measured and analysed in all studied springs.

	**Auvergne-France**								**Sardinia-Italy**							
Variable/Spring	PSAL	FDBL	BARD2	LEFO	CERI	CHAT	BENE	POIX1	SGA	CAD1	CAD2	SSA	ABB	SBM	SMA	CAS
T (°C)	20.7	18.0	14.2	32.6	18.1	27.3	14.7	13.3	29.3	49.3	53.0	20.9	22.1	32.2	11.2	71.5
pH	6.89	6.42	6.53	7.02	6.58	7.15	7.06	8.25	7.30	7.91	7.98	8.99	6.32	9.33	6.50	7.06
Conductivity (μS cm^−1^at 25 °C)	8540	1344	6510	4230	4040	8440	4120	4810	590	1152	1193	930	5270	1020	4890	8890
Na^+^ (mg L^−1^)	1628	23	930	920	610	917	689	763	26	220	231	170	1539	178	804	1208
K^+^ (mg L^−1^)	179.9	6.3	203.8	56.3	126.3	110.3	127.9	72.9	1.7	5.3	1.6	2.3	36.2	2.1	62.3	42.1
Mg^2+^ (mg L^−1^)	132.0	49.1	139.9	36.7	104.4	426.3	152.2	75.0	30.4	5.0	2.0	<D.L.	83.0	<D.L.	340.8	334.1
Ca^2+^ (mg L^−1^)	327	237	5	91	178	757	158	164	44	8	6	12	16	12	80	56
HCO_3_^−^ (mg L^−1^)	2840	650	2013	1850	1870	2200	1910	610	274.5	29.5	29.3	17.1	3100	20.7	3044	30.5
F^−^(mg L^−1^)	1.1	0.4	0.8	6.3	1.6	0.9	0.4	0.2	0.1	6.6	7.2	7.8	0.7	4.0	<D.L.	1.8
Cl^−^ (mg L^−1^)	1752	44	784	296	445	2419	293	1160	46	284	305	220	432	269	262	2794
Br^−^ (mg L^−1^)	5.1	<D.L.	4.0	1.4	0.2	5.3	0.1	2.0	0.4	0.7	0.7	0.5	0.9	0.7	<D.L.	8.2
SO_4_^2−^ (mg L^−1^)	113	69	43	332	30	403	228	553	10	42	43	39	405	34	219	89
N-NH_4_^+^(μ g N L^−1^)	2.38	0.32	1.20	1.11	1.14	0.77	0.85	0.33	8	47	49	2	<D.L.	50	<D.L.	27
N-NO_2_^−^ (μ g N L^−1^)	<D.L.	<D.L.	0.02	0.01	0.01	0.02	0.02	0.01	6	1	1	1	1	<D.L.	6	1
N-NO_3_^−^ (μ g N L^−1^)	255	32029	386	662	506	130	704	210	2448	567	14	9	7	<D.L.	110	81
total nitrogen (μ g N L^−1^)									3743	1562	606	1867	734	1685	1823	700
reactive phosphorus (μ g P L^−1^)									16	361	22	7	246	22	27	21
total phosphorus (μ g P L^−1^)									37	441	97	79	607	106	273	37

## Results

### Environmental variables

The results of the physical and chemical variables for all springs are presented in Table 2. The sites showed heterogeneity in temperature, pH and conductivity values, with wider ranges for Sardinia than Auvergne. Springs with temperatures <20 °C were FDBL, BARD2, CERI, BENE, and POIX1 in Auvergne, and SMA in Sardinia. The highest temperature values were observed at LEFO (32.6 °C) and CHAT (27.3 °C) in Auvergne, and at CAD1 (49.3 °C), CAD2 (53 °C) and CAS (71.5 °C) in Sardinia. pH ranged from slightly acid to alkaline with the minimum and maximum values at FDBL (6.42) and POIX1 (8.25) in Auvergne, and at ABB (6.32) and SBM (9.33) in Sardinia, respectively. Conductivity ranged from 1344 µS cm^−1^ at FDBL to 8540 µS cm^−1^ at PSAL in Auvergne, and from 590 µS cm^−1^ at SGA to 8890 µS cm^−1^ at CAS in Sardinia. In Auvergne, HCO_3_^−^ was the most abundant ion at PSAL, FDBL, BARD2, LEFO, CERI and BENE, followed by Na^+^ and Cl^−^ at PSAL, BARD2, CERI and BENE, Na^+^ and SO_4_^2−^ at LEFO, and Ca^2+^ and SO_4_^2−^ at FDBL. Additionally, HCO_3_^−^, Na^+^ and Cl^−^ were the main ions at CHAT and POIX1, but Cl^−^ was the most abundant. In Sardinia, HCO_3_^−^ was the most abundant ion at ABB, SMA and SGA, whereas Cl^−^ was the most abundant ion at CAS, CAD1, CAD2, SBM and SSA. The other most abundant ions were Na^+^ (except at SGA), SO_4_^2−^ and Mg^2+^ at ABB, CAS and SMA, and Ca^2+^ at SMA, CAS and SGA. Overall, N-NO_3_^−^ values were higher in springs in Auvergne (130–32029 µg L^−1^) than in Sardinia (<D.L.-2448 µg L^−1^). In Auvergne, the maximum value was recorded at FDBL, and in Sardinia at SGA. The reactive and total phosphorus, available only for springs in Sardinia, showed higher values at ABB (246 and 607 µg P L^−1^, respectively) and at CAD1 (361 and 441 µg P L^−1^, respectively).

**Figure 2:**
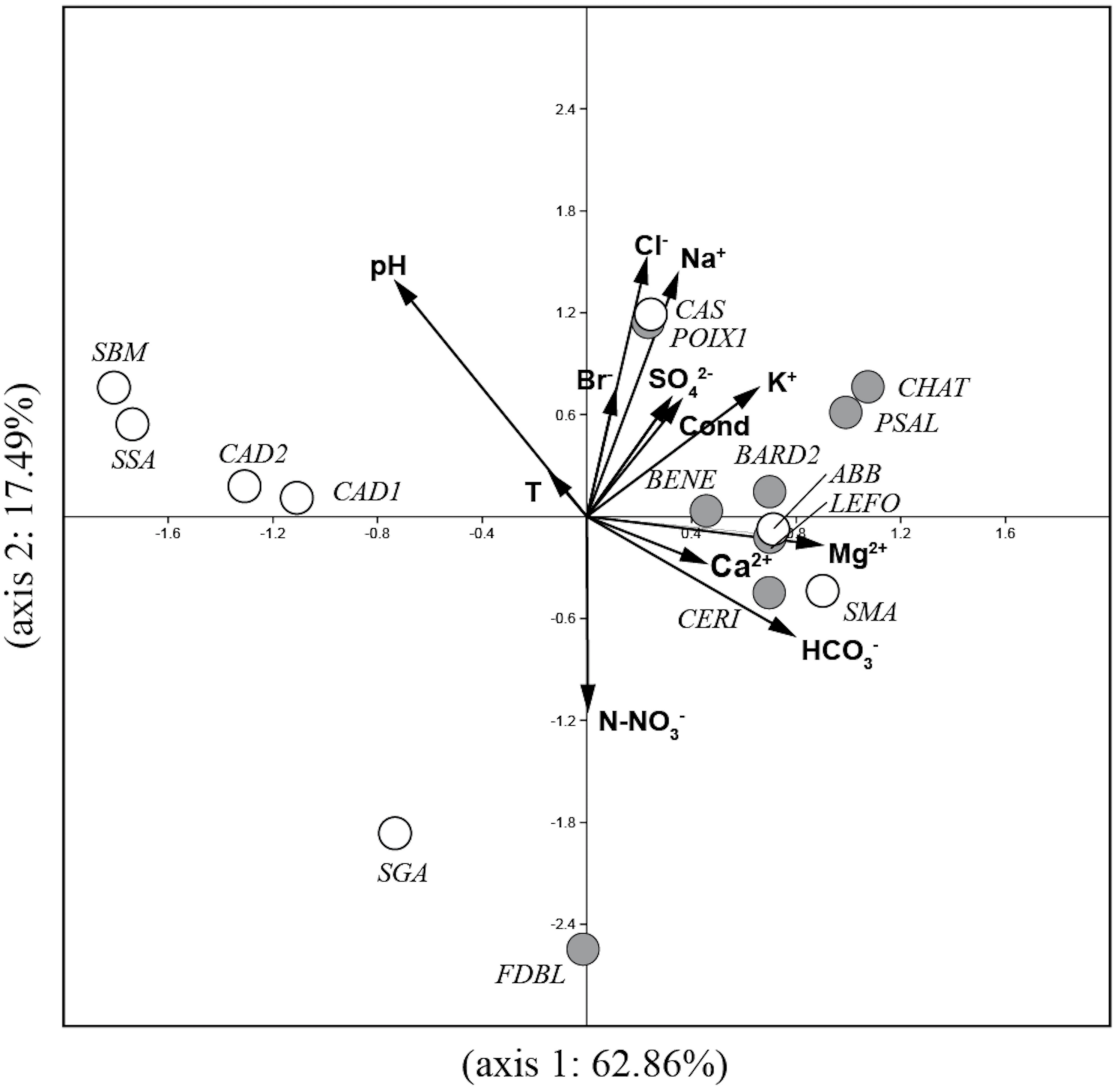
Plot of the PCA analysis performed on environmental variables for all studied springs. Gray circles = sites in Auvergne; white circles = sites in Sardinia. All sigles of the sites are reported in Table 1.

The PCA analysis (Fig. 2) performed on environmental variables explained 80.4% of the variance in the data in the first two axes (62.9%: axis 1 and 17.5%: axis 2). The ordination of data distinguished four main groups of springs: 1) six springs in Auvergne and ABB and SMA in Sardinia associated with Mg^2+^, Ca^2+^, HCO_3_^−^ and K^+^; 2) POIX1 in Auvergne and CAS in Sardinia associated with Na^+^ and Cl^−^; 3) CAD1, CAD2, SSA and SBM in Sardinia associated with temperature and pH and 4) FDBL in Auvergne and SGA in Sardinia associated with N-NO_3_^−^.

### Diatom assemblages

In total, 207 diatom taxa from 59 genera were found, of which 61 (23 genera) in the springs of Auvergne, and 178 (55 genera) in the springs of Sardinia.

The species common to springs in Auvergne and Sardinia were 24 from 13 genera (11.6% of the total species) and included several *Navicula* (e.g., *N. cincta* (Ehrenberg) Ralfs, *N. veneta* Kützing, *N. sanctamargaritae* Beauger, *N. vilaplanii* (Lange-Bertalot & Sabater) Lange-Bertalot & Sabater) and *Nitzschia* (e.g., *N.* aff. *liebethruthii*, *N. linearis* W. Smith, *N. inconspicua* Grunow and *N. microcephala* Grunow). Other common species were *Crenotia thermalis* (Rabenhorst) Wojtal, *Denticula subtilis* Grunow, *Diploneis elliptica* (Kützing) Cleve, *Halamphora normanii* (Rabenhorst) Levkov, *Gomphonema parvulum* Kützing, *Tryblionella debilis* Arnott ex O’Meara and *T. hungarica* (Grunow) Frenguelli.

Assemblages from Auvergne were composed of 12 taxa abundant (RA ≥5%), 8 taxa frequent (RA >1–5%) and 48 taxa rare (RA ≤1%). The most species-rich genera were *Nitzschia* and *Navicula* with 15 and six taxa, respectively. The most common species were *Crenotia thermalis* (in all sites except POIX1), *Planothidium frequentissimum* (Lange-Bertalot) (in six of the eight springs), *Navicula sanctamargaritae* and *N. veneta* (in four of the eight springs).

Assemblages from Sardinia were composed of five taxa abundant (RA ≥5%), 11 taxa frequent (RA >1–5%) and 163 taxa rare (RA ≤1%). The most species-rich genera were *Nitzschia*, *Navicula* and *Gomphonema* with 27, 19 and 10 taxa respectively. The most common species were *Navicula veneta* (in all sites), *Nitzschia amphibia* Grunow (in seven of the eight springs), *Lemnicola exigua* (Grunow) Kulikovskiy, Witkowski & Pliński, *Cocconeis euglypta* Ehrenberg, *Halamphora normanii* (Rabenhorst) Levkov, *Nitzschia inconspicua* and *Rhopalodia operculata* (C. Agardh) Håkansson (in six of the eight springs).

A floristic list of all taxa with a minimum RA >1% in all samples, along with their ecological preferences, is reported in Table 3. These species are mainly linked to the aquatic environment, with preferences for alkaline and fresh-brackish waters and high nutrient contents.

**Table 3:** Floristic list of all diatom taxa with relative abundance >1% and ecological preferences for pH reaction, salinity, trophic state and moisture according to Van Dam, Mertens & Sinkeldam (1994).

		**Auvergne-France**								**Sardinia-Italy**											
Taxa	Sigles	PSAL	FDBL	BARD2	LEFO	CERI	CHAT	BENE	POIX1	SGA	CAD1	CAD2	SSA	ABB	SBM	SMA	CAS	pH reaction	S	TS	M
*Achnanthidium minutissimum* (Kützing) Czarnecki	ADMI									1.1	0.5	2.4	0.9		3.3			n	f-b	hyper	3
*Aulacoseira granulata* (Ehrenberg) Simonsen	AUGR										0.6		0.3				19.2	ak	f-b	eu	1
*Cocconeis euglypta* Ehrenberg	CEUG									0.1	3.1	4.2	2.2		0.2		0.6	ak	f-b	eu	2
*Crenotia thermalis* (Rabenhorst) Wojtal	CRTH	68.2	0.2	49.9	96.2	45.8	96.8	38.3						4.5	0.1	6.3		n	b-f		
*Encyonema silesiacum* (Bleisch) D.G. Mann	ESIL												25.9					ak	f-b	hyper	1
*Fallacia pygmea (Kützing) Stickle & D.G. Mann*	FPYG			3.1																	
*Fallacia* sp.	FALS			1.2																	
*Lemnicola exigua* (Grunow) Kulikovskiy, Witkowski & Pliński	LEXI									0.6	0.4	0.9	2.0		44.6		0.1	ak	f-b	oligo-eu	3
*Navicula gregaria* Donkin	NGRE								29.6									ak	b-f	eu	3
*Navicula salinarum Grunow*	NSAL								9.0									n	b	eu	1
*Navicula sanctamargaritae* A. Beauger	NYSG	30.0		12.5		30.3		55.4						20.4		34.5					
*Navicula veneta* Kützing	NVEN			1.2		1.2	0.2		6.3	0.4	1.0	1.3	4.5	4.6	0.9	2.7	0.2	ak	f-b	eu	3
*Navicula vilaplanii* (Lange-Bertalot & Sabater) Lange-Bertalot & Sabater	NVIP																				
*Nitzschia* aff. *bulnheimiana*	N. aff. bul								18.0												
*Nitzschia amphibia* Grunow	NAMP									19.0	2.3	7.2	0.2	0.1	1.0		0.2	ak	b	eu	1
*Nitzschia* aff. *liebetrhutii*	N. aff. lieb																7.2				
*Nitzschia inconspicua* Grunow	NINC									1.7	8.9	2.3	1.1	1.7	8.1			ak	b-f	eu	3
*Nitzschia microcephala* Grunow in Cleve & Möller	NMIC										1.5	0.5	3.2		2.3		1.5	ak	f-b	eu	1
*Nitzschia palea* (Kützing) W. Smith	NPAL										19.3	0.5	0.4		0.9		0.5	n	f-b	hyper	3
*Nitzschia supralitorea* Lange-Bertalot	NZSU			3.1																	
*Pinnularia kuetzingii* Krammer	PKUT			0.5	3.5		1.7														
*Pinnularia* sp.3	PINS													9.4		33.8					
*Planothidium frequentissimum* (Lange-Bertalot) Lange-Bertalot	PLFR		1.4	10.3		21.7	0.2	4.4	2.4	0.5			6.0	32.7	2.2			ak	f-b	oligo-eu	
*Planothidium lanceolatum* (Brébisson ex Kützing) Lange-Bertalot	PTLA		61.1															ak	f-b	eu	3
*Planothidium victorii* P.M. Novis, J. Braidwood & C. Kilroy	PVIC					0.2		1.9													
*Pseudostaurosira bardii* Beauger, C.E. Wetzel & Ector	PBAR			3.4																	
*Pseudostaurosira brevistriata* (Grunow) D.M. Williams & Round	SBRE										0.6	5.6					1.2	ak	f-b	oligo-eu	2
*Rhopalodia operculata* (C. Agardh) Håkansson	ROPE										0.9	0.6	1.9	5.4		5.4	3.1				
*Sellaphora labernardierei* A. Beauger, C.E.Wetzel & L. Ector	SLAB		31.5	0.7																	
*Sellaphora nigri* (De Notaris) C.E. Wetzel & L. Ector	SNIG		1.0	7.0						52.8	0.2	1.5	1.4								
*Sellaphora saugerresii* (Desmazières) C.E. Wetzel & D.G. Mann	SSGE									4.1				8.3							
*Stephanodiscus neoastraea* Håkansson & Hickel	SNEO										3.7	0.8					14.8	akb	f-b	eu	1
*Tryblionella apiculata* Gregory	TAPI								12.1												
*Tryblionella hungarica* (Grunow) D.G. Mann	THUN								13.6									ak	b-f	eu	1
*Tryblionella* sp.	TRYS								3.2												
*Ulnaria ulna* (Nitzsch) Compère	UULN									1.5	3.1	1.8			0.1		0.8	ak	f-b	oligo-eu	1

**Notes.**

Preferences for pH reaction ak alkaliphilous n circumneutral

preferences for Salinity f-b fresh-brackish b brackish b-f brackish-fresh

preferences for Trophic State (species characteristic of) oligo oligotrophic environments eu eutrophic environments hyper hypereutrophic environments

preferences for Moisture 1 never, or only very rarely, occurring outside water bodies 2 mainly occurring in water bodies, sometimes on wet places 3 mainly occurring in water bodies, also rather regularly on wet and moist places 4 mainly occurring on wet and moist or temporarily dry places 5 nearly exclusively occurring outside water bodies

**Figure 3:**
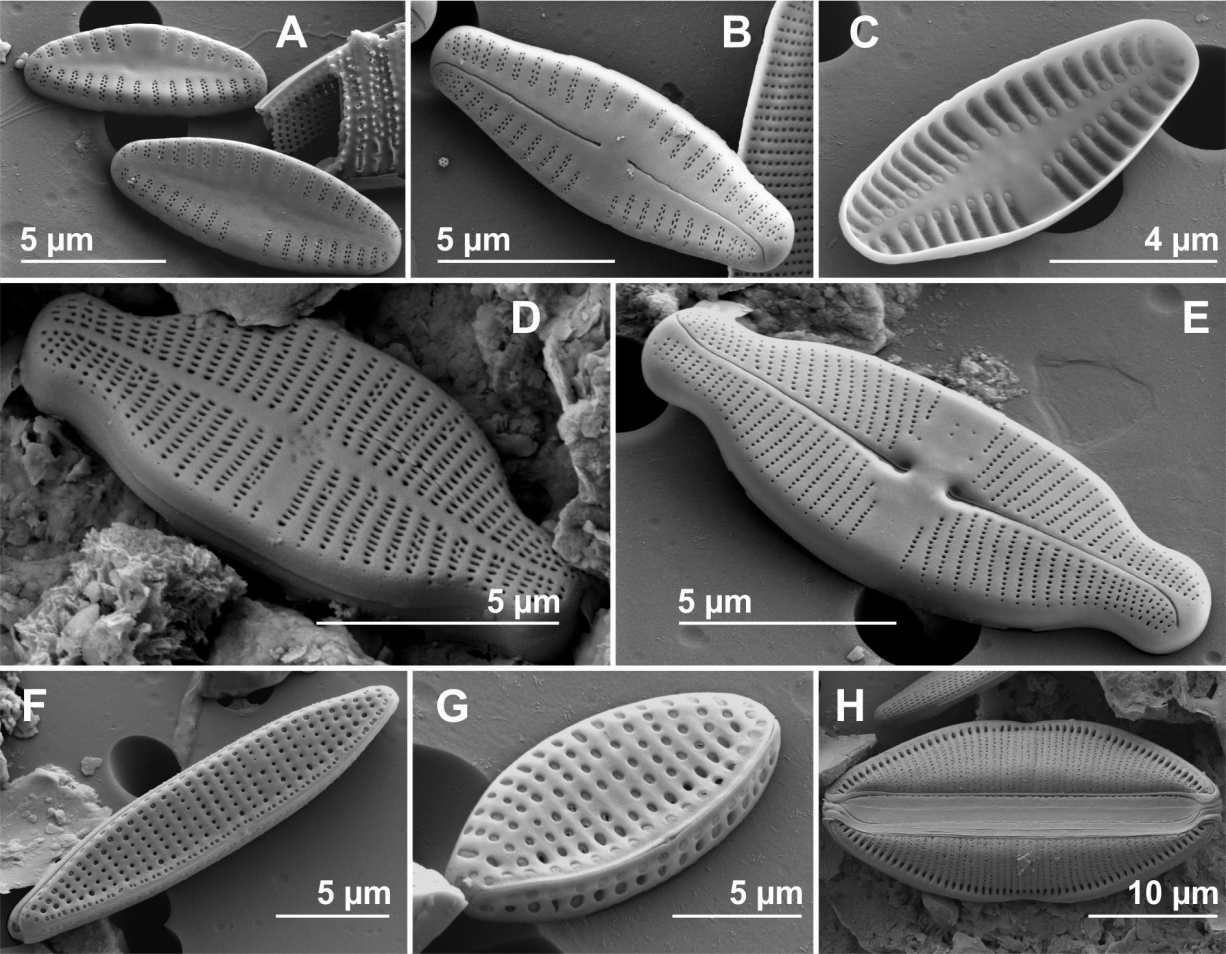
Scanning electron microscopy (SEM). (A) *Crenotia thermalis*, external rapheless valve view. (B) *Crenotia thermalis*, external raphe valve view. (C) *Crenotia thermalis*, internal rapheless valve view. (D) *Lemnicola exigua*, external rapheless valve view. (E) *Lemnicola exigua*, external raphe valve view. (F) *Nitzschia amphibia*, external valve view. (G) *Nitzschia inconspicua*, external valve and mantle view. (H) *Rhopalodia operculata*,** external frustule view.

Some abundant and common species at several sites, considered more representative for the studied springs in Auvergne and Sardinia, were illustrated using scanning electron microscopy (Figs. 3A–3H).

The results of the structural indices are reported in Table 4. In Auvergne, species richness, was lower at LEFO and higher at BARD2. The minimum and maximum values of the Shannon-Wiener index were recorded at LEFO and POIX, respectively, and the minimum and maximum values of the Pielou’s evenness index at CHAT and POIX1, respectively. In Sardinia, the minimum and maximum values of species richness, Shannon-Wiener index and Pielou’s evenness index were found at SMA and CAD2, respectively.

**Table 4:** Values of species richness (SR), Shannon–Wiener diversity index (H′) and Pielou’s evenness index (J′) for diatom assemblages of Auvergne and Sardinia.

	**SR**	**H′**	**J**
**Auvergne-France**			
PSAL	8	1.05	0.35
FDBL	15	1.36	0.35
BARD2	25	2.70	0.58
LEFO	3	0.24	0.15
CERI	8	1.68	0.56
CHAT	7	0.27	0.09
BENE	4	1.31	0.65
POIX1	20	3.00	0.69
**Sardinia-Italy**			
SGA	26	2.48	0.53
CAD 1	52	4.28	0.75
CAD 2	72	5.22	0.85
SSA	38	3.45	0.66
ABB	15	2.44	0.64
SBM	35	2.79	0.56
SMA	6	0.93	0.38
CAS	43	3.84	0.71

### Differences among assemblages and relationships with environmental variables

**Figure 4:**
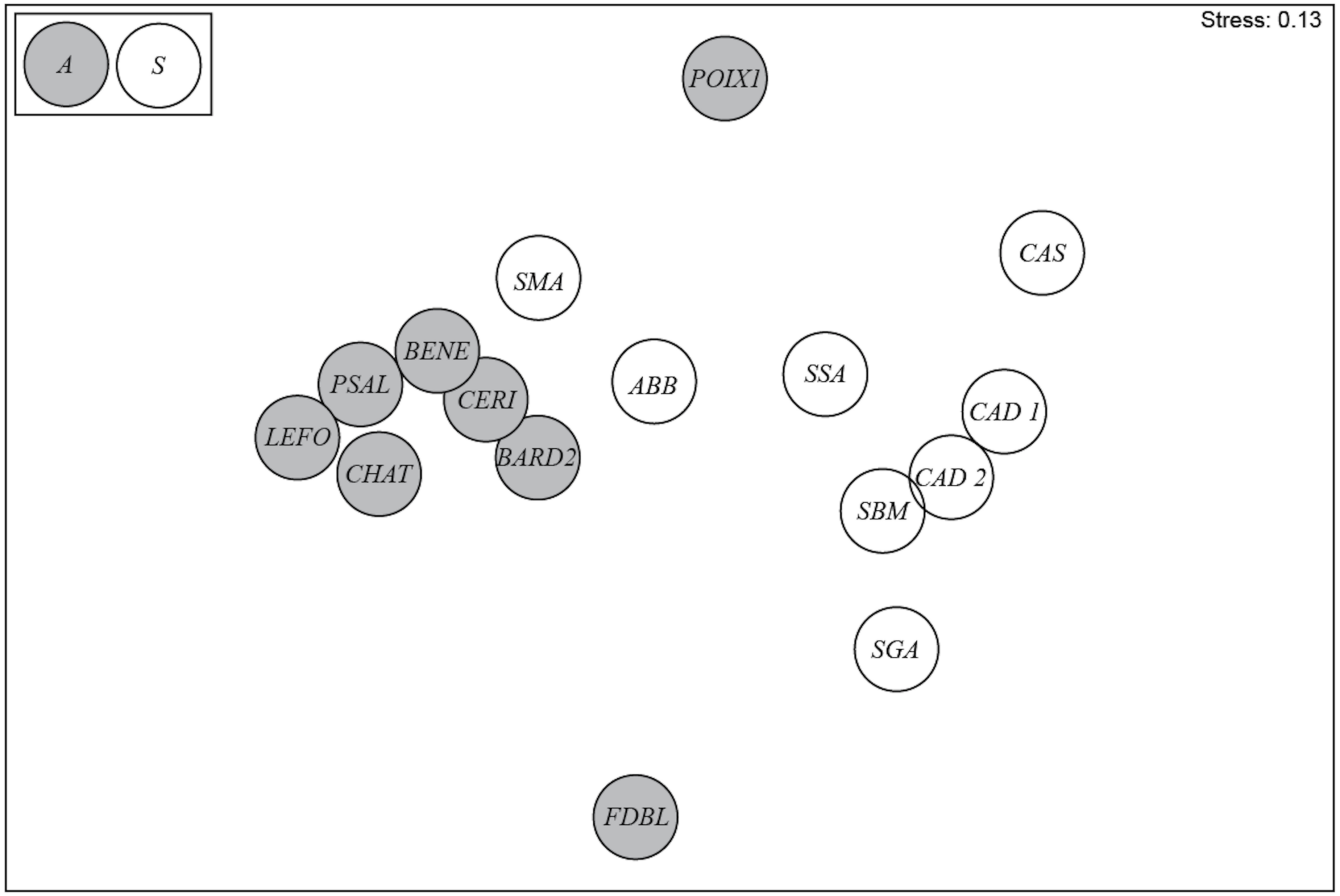
nMDS ordination plot for springs in Auvergne and Sardinia. Gray circles = sites in Auvergne; white circles = sites in Sardinia. All sigles of the sites are reported in Table 1.

The diatom assemblages from springs in Auvergne and Sardinia were clearly separated in the nMDS ordination plot (Fig. 4). Significant differences between the two groups were confirmed by the ANOSIM test (global *R* = 0.516; *p* = 0.002). According to SIMPER analysis, the average dissimilarity was 93.49%. The greatest contribution to the differences was provided by *Crenotia thermalis* (33.34%) and *Navicula sanctamargaritae* (11.64%) (Table 5). The assemblages from Auvergne revealed a higher average similarity among sites (33.98%) than in assemblages from Sardinia (13.01%). The greatest contribution to the differences was provided by *Crenotia thermalis* (81.63%) for the sites in Auvergne, and *Nitzschia inconspicua* (14.86%), *Navicula veneta* (11.81%) and *Rhopalodia operculata* (10.33%) for the sites in Sardinia (Table 5).

**Table 5:** Results of the SIMPER analysis (taxa that most contributed to dissimilarity among diatom assemblages between Auvergne and Sardinia and taxa that most contributed to similarity among diatom assemblages within each geographic region with their percentage contribution at the cut-off level of 70%).

Taxons	Contribution%	Contribution cumulative %
**Auvergne vs Sardinia (average dissimilarity 93.49%)**		
*Crenotia thermalis*	33.3	33.3
*Navicula sanctamargaritae*	11.6	45.0
*Planothidium lanceolatum*	5.3	50.3
*Planothidium frequentissimum*	5.2	55.4
*Sellaphora nigri*	4.7	60.1
*Lemnicola exigua*	4.1	64.2
*Pinnularia* sp. 3	3.2	67.4
*Sellaphora labernardierei*	2.8	70.2
**Auvergne (averagesimilarity 33.98%)**		
*Crenotia thermalis*	81.6	81.6
**Sardinia (average similarity13.01%)**		
*Nitzschia inconspicua*	14.9	14.9
*Navicula veneta*	11.8	26.7
*Rhopalodia operculata*	10.3	37.0
*Nitzschia amphibia*	8.8	45.8
*Cocconeis euglypta*	6.7	52.5
*Navicula sanctamargaritae*	6.6	59.8
*Nitzschia microcephala*	6.5	65.5
*Achnanthidium minutissimum*	4.8	70.3

**Figure 5:**
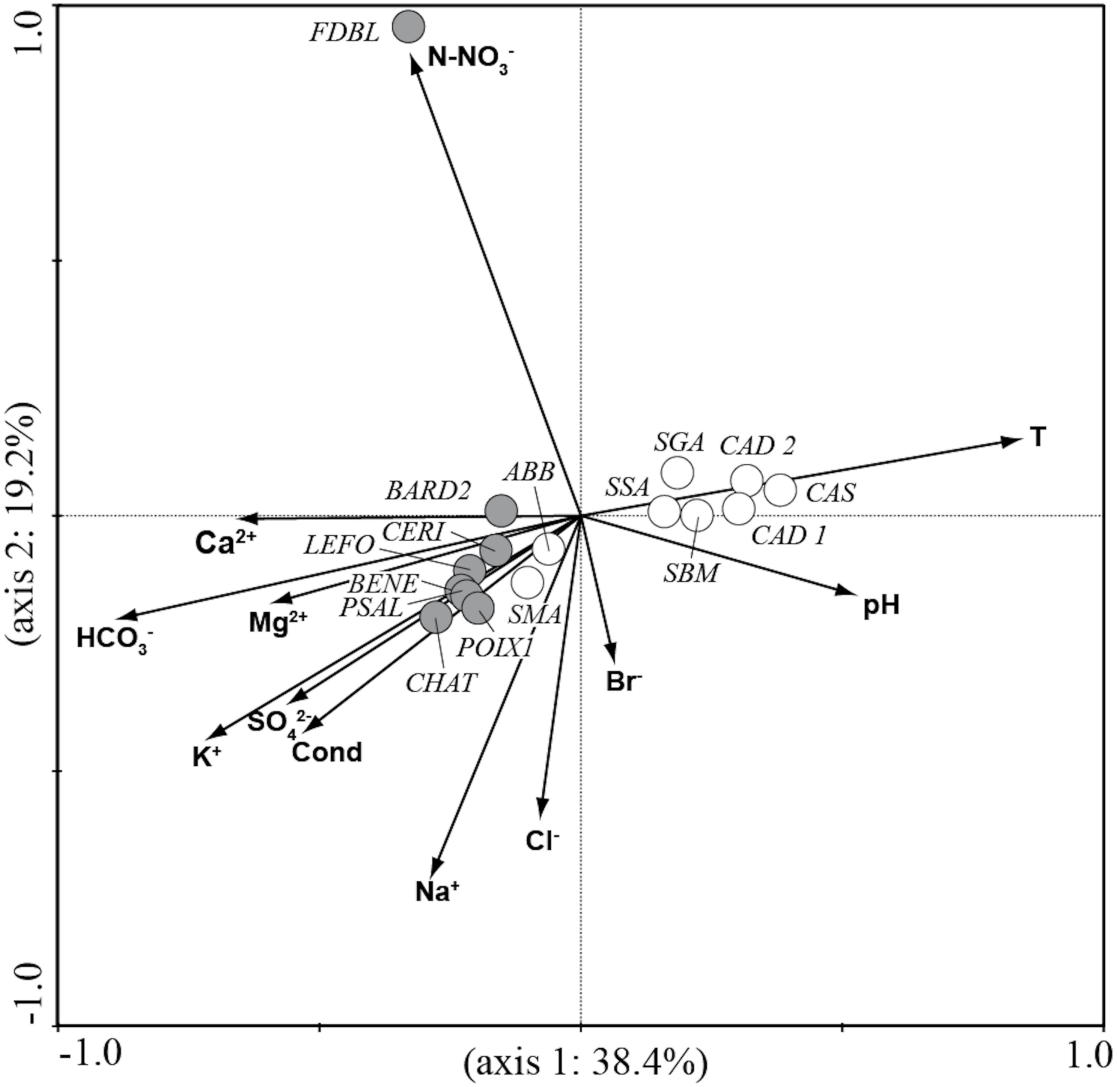
CCA ordination plot for the sites. Vectors, environmental variables; gray circles, sites in Auvergne; white circles, sites in Sardinia. All sigles of the sites are reported in Table 1.

**Figure 6:**
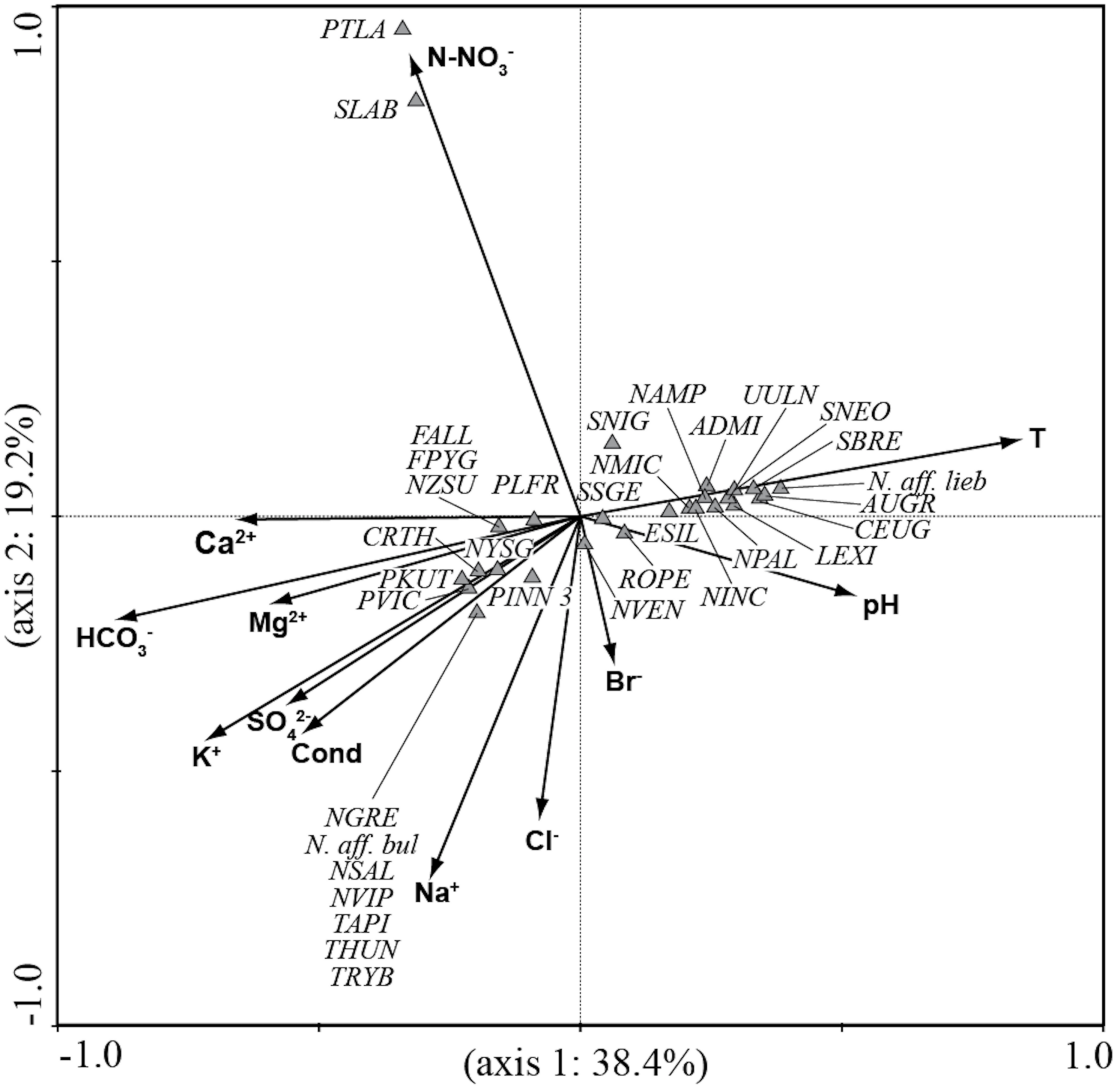
CCA ordination plot for diatom species. Vectors, environmental variables; triangles, diatom species. All sigles of the species are reported in Table 3.

CCA analysis (Fig. 5 and Fig. 6) explained 58.2% of the variance in the data in the first two axes (axis 1: 19.8% and axis 2: 38.4%). Ordination data distinguished three main groups of species: (1) species like *Aulacoseira granulata* (Ehrenberg) Simonsen, *Cocconeis euglypta*, *Encyonema silesiacum* (Bleisch) D.G. Mann, *Lemnicola exigua*, *Nitzschia inconspicua*, *Nitzschia microcephala*, *Nitzschia palea* (Kützing) W. Smith, from six springs of Sardinia (SGA, SSA, SBM, CAD1, CAD2, CAS) associated with water temperature and pH; (2) species like *Crenotia thermalis*, *Navicula gregaria* Donkin, *N. salinarum* Grunow, *Nitzschia* aff. *liebethruthii*, *Pinnularia kuetzingii* Krammer, *Pinnularia* sp. 3, *Tryblionella apiculata* W. Gregory and *T*. *hungarica* from seven springs of Auvergne (CHAT, POIX1, PSAL, BENE, LEFO, CERI, BARD2) and two springs of Sardinia (ABB and SMA) associated with various ions and conductivity; (3) *Planothidium lanceolatum* (Brébisson ex Kützing) Lange-Bertalot and *Sellaphora labernardierei* Beauger, C.E. Wetzel & Ector from FDBL in Auvergne associated with higher values of N-NO_3_^−^. Overall, pH (*p* = 0.0060), conductivity (*p* = 0.0120) and HCO_3_^−^ (*p* = 0.0020) were the most significant variables for diatom assemblages.

## Discussion

### Structure of the diatom assemblages

Diatoms were found in all investigated sites, including those with the highest water temperature and mineralization, factors generally considered not very conducive for diatom growth (e.g., Patrick, 1948; Mandal & Sarkar, 2015; Żelazna Wieczorek, Olszyński & Nowicka-Krawczyk, 2015). Our observations are supported by studies carried out in different geographic areas, such as Africa (Mpawenayo, Cocquyt & Nindorera, 2005), Azores (Quintela et al., 2013), the Czech Republic (Kaštovský & Komárek, 2001), France (Beauger et al., 2017), Italy (Andreoli & Rascio, 1975), Russia (Nikulina & Kociolek, 2011) and Thailand (Pumas, Pruetiworanan & Peerapornpisal, 2018), that reported diatom assemblages at temperatures >50 °C. Several studies also described diatom assemblages in springs with high mineral content (conductivity from 4580 to 18340 µS cm^−1^) (Mpawenayo, Cocquyt & Nindorera, 2005; Beauger et al., 2017; Angel et al., 2018). In Auvergne, species richness, diversity and evenness were generally higher in springs with lower temperature (FDBL, POIX1 and BARD2), and lower in several springs with high mineralization (PSAL, LEFO, CERI, CHAT, and BENE). In Sardinia, the values of these structural indices were generally higher in two warmer springs (CAD1 and CAD2) and, as in Auvergne, lower in two more mineralized springs (SMA and ABB). Our results are, although partially in contrast, in good agreement with those reported in literature. For example, springs in Galicia (Spain) had a higher species richness in hot springs than in cold springs, and only seven taxa were found in a cold-water spring with moderate mineralization (Leira, Meijide-Failde & Torres, 2017). Furthermore, in thermo-mineral springs in Azores, the highest values of diversity were recorded in a cold and in a thermal site (17–58 °C) (Quintela et al., 2013). This suggests that different combinations of water temperature and mineralization can support differently structured assemblages. The values of the structural indices observed at CAS, although unusually high, were lower than the two other warmer springs CAD1 and CAD2. This seems in accordance with the results of other studies that have reported an impoverishment of species at temperatures >70 °C (e.g., Nikulina & Kociolek, 2011; Quintela et al., 2013). A previous study at CAS also showed a lower species richness in assemblages collected close to the water emergence point, where the temperature reaches 70 °C (Lai et al., 2019).

The high number of species found in this study, especially in Sardinia, suggests wide spatial heterogeneity and variability of environmental conditions. In fact, these factors generate high habitat diversity and high total species richness in springs, according to Cantonati et al. (2012a).

In Sardinia, the high geodiversity (Fiorentino, Curioni & Pisano, 2017), probably plays an important role in the richness of diatom flora. A low number of species was found only at SMA. This is consistent with the results reported for limnocrenic and iron springs in the southeastern Alps (Cantonati et al., 2012b). In Auvergne, numerous sites showed a low number of species. The lower values found at LEFO, BENE and CHAT suggest a possible role of factors such as morphological alteration and water abstraction. In fact, these factors cause an impoverishment of the biota (e.g., Cantonati et al., 2012a).

### Species composition in the two geographical regions

In Auvergne, the most abundant species included *Crenotia thermalis*, *Navicula sanctamargaritae*, *Planothidium frequentissimum* and *Sellaphora labernardierei*, common to several sites, and *Navicula gregaria* and *Planothidium lanceolatum* observed at single sites.

*Crenotia thermalis* was observed in almost all sites. This species, occasionally encountered in running waters, is characteristic of electrolyte-rich inland habitats, particularly thermal and mineral springs (Wojtal, 2013; Lange-Bertalot et al., 2017). *Navicula sanctamargaritae* was observed at PSAL, BARD2, CERI and BENE, with high mineralization levels of water, mainly in association with *Crenotia thermalis*. *Navicula sanctamargaritae* was first described in the Massif Central, in a highly mineralized thermo-mineral spring (Beauger et al., 2015). *Planothidium frequentissimum* was found at most sites, except LEFO and PSAL, respectively characterized by higher temperature and conductivity. According to Van Dam, Mertens & Sinkeldam (1994), this taxon has a wide ecological range. *Sellaphora labernardierei* was found at FDBL, where it was recently described (Beauger et al., 2016) and at BARD2. At FDBL, enriched with calcium and especially nitrates, *Sellaphora labernardierei* dominated with *Planothidium lanceolatum*, observed only at this site. *Planothidium lanceolatum* was reported as a sensitive indicator of calcium ions and nutrient enrichment in Polish springs (Wojtal, 2013). At BARD2, *Sellaphora labernardierei* was present with lower abundance, associated with *Pseudostaurosira bardii*. This latter species, recently described from this spring (Beauger et al., 2018), was observed only at this site which is characterized by high conductivity and higher concentrations of sodium, chloride and bicarbonate. *Navicula gregaria* was observed at POIX1, together with *Navicula salinarum*, *Nitzschia* aff *bulnheimiana* and several *Tryblionella* species. All these taxa were found only at this site which is characterized by high chloride content and degassing of hydrogen sulphide. *Navicula gregaria* was found on coasts and inland salt springs, and *N. salinarum* in inland brackish waters, including athalassic ecosystems (Lange-Bertalot, 2001; Żelazna Wieczorek, Olszyński & Nowicka-Krawczyk, 2015). Unlike *Navicula gregaria* that was observed in springs of different countries (Owen, Renaut & Jones, 2008; Leira, Meijide-Failde & Torres, 2017; Angel et al., 2018), *N. salinarum* is not classically encountered in springs. However, it was found in the salt springs of the Nidziańka Basin in Poland (Wojtal, 2013). *Tryblionella apiculata* and *T. hungarica* were observed in salt-rich inland habitats (Lange-Bertalot et al., 2017). In Chile, *Tryblionella hungarica* was found occasionally also in freshwater fumeroles (Angel et al., 2018).

In Sardinia, the most abundant species included *Lemnicola exigua*, *Navicula sanctamargaritae*, *Pinnularia* sp. 3, *Planothidium frequentissimum* and *Sellaphora nigri* (De Notaris) C.E. Wetzel & Ector, common to several sites, and *Encyonema silesiacum* (De Notaris) C.E. Wetzel & Ector, observed at a single site. Among these, *Lemnicola exigua*, *Planothidium frequentissimum* and *Encyonema silesiacum* were previously observed in a karst spring of the Island (Lai et al., 2016; Lai et al., 2018). *Lemnicola exigua* was observed at all springs except ABB and SMA, which were characterized by cooler waters enriched with sodium and bicarbonate. This species, reported in geothermal springs of several geographic areas (e.g., López-Sandoval et al., 2016; Angel et al., 2018; Pumas, Pruetiworanan & Peerapornpisal, 2018), has a wide ecological range (Van Dam, Mertens & Sinkeldam, 1994; Lange-Bertalot et al., 2017). *Navicula sanctamargaritae*, as in Auvergne, was observed in sites with high conductivity (ABB and SMA) in association with *Crenotia thermalis*. *Navicula sanctamargaritae* dominated with *Planothidium frequentissimum* in ABB and with *Pinnularia* sp. 3 in SMA. As in Auvergne, *Planothidium frequentissimum* was not found at sites with higher temperature (CAD1, CAD2 and CAS). *Sellaphora nigri* is also a species with a wide ecological range, but was generally more abundant in degraded habitats according to Lange-Bertalot et al. (2017). This species was observed at CAD1, CAD2 and SSA. As in Auvergne, *Sellaphora nigri* was found in the presence of a higher concentration of nitrates at SGA, where it dominated with *Nitzschia amphibia*. *Encyonema silesiacum* was recorded only at SSA, characterized by medium-high conductivity and low nutrient content. This species, common to habitats less disturbed by human activities, can tolerate nutrient enrichment (Lange-Bertalot et al., 2017), and is hypereutraphentic according to Van Dam, Mertens & Sinkeldam (1994). *Encyonema silesiacum* also prefers low-medium electrolyte content (Lange-Bertalot et al., 2017) and was reported as a species indifferent to salinity in thermal springs of the far east, in Russia (Nikulina & Kociolek, 2011). Other abundant species included *Nitzschia* aff. *liebethruthii*, *N. inconspicua*, *N. palea*, *Pseudostaurosira brevistriata* (Grunow) D.M. Williams, *Rhopalodia operculata* and *Sellaphora saugerresii* (Desmazières) C.E. Wetzel & D.G. Mann. Most of these species have been reported in thermo-mineral systems by several authors (e.g., Villeneuve & Pienitz, 1998; Mpawenayo & Mathooko, 2004; Mannino, 2007; Quintela et al., 2013; Bhakta, Das & Adhikary, 2016; Angel et al., 2018). Centric diatoms like *Aulacoseira granulata* and *Stephanodiscus neoastraea* were found at CAD1, SSA and CAS, and were more abundant at CAS. These species, rarely found in spring environments, prefer fresh-brackish waters according to Van Dam, Mertens & Sinkeldam (1994). However, the higher abundance at CAS suggests a wider tolerance of these species for salinity with respect to the range indicated by Van Dam, Mertens & Sinkeldam (1994). Moreover, *Aulacoseira granulata* was found in high water temperatures and mineralization levels in springs of the far east in Russia (Nikulina & Kociolek, 2011).

### Comparison among diatom assemblages

As revealed by nMDS and SIMPER analyses, diatom assemblages showed low similarity among sites within each geographic region, and high dissimilarity level among sites in Auvergne and Sardinia. In Auvergne, FDBL and POIX1 resulted well separated between them and from a cluster formed by the remaining six sites. The differences in diatom assemblages of these two springs were probably due to the higher concentrations of several ions at POIX1 and of nitrates at FDBL, indicating a high human impact, mainly due to agricultural activities in the surrounding area (Beauger et al., 2016). The major contribution to similarity among sites was given by *Crenotia thermalis*. This taxon dominated at all six springs but was not found at POIX1, and only one specimen was observed at FDBL.

In Sardinia, the similarity among sites was lower than in Auvergne. SMA, ABB, SSA, CAS and SGA resulted well separated among them and from a cluster formed by the remaining three sites. CAD1, CAD2 and SBM showed more similar environmental conditions as a result of a probable single hydrogeological circuit (Dettori, Zanzari & Zuddas, 1982). Their diatom assemblages were composed of several common species with quite similar abundances. In this same geothermal area, the different species composition at SSA could be attributed to lower water temperature and conductivity, but also lower size, discharge and water current velocity.

In general, differences in diatom assemblages among sites of Sardinia seemed mainly associated with differences in water temperature, pH and ionic composition.

The assemblages of Auvergne and Sardinia formed two clearly separated clusters. They differed for several species, but a greater contribution was due to *Crenotia thermalis* and *Navicula sanctamargaritae*, more widely distributed in springs in Auvergne than in Sardinia. These results highlighted a wide spatial heterogeneity in the distribution of species. This variability is not surprising since several studies reported different richness and species composition patterns among thermo-mineral systems both on the local and regional scale (e.g., Mpawenayo & Mathooko, 2004; Owen et al., 2004; Owen, Renaut & Jones, 2008). Local factors such as water temperature, light, substratum particle size, discharge, water current velocity and water chemistry are considered important drivers for growth and distribution of diatom species (Patrick, 1948; Cantonati, Gerecke & Bertuzzi, 2006). For example, observations on diatom flora from many cold springs in Sardinia highlighted the importance of hydrogeological conditions in the variations of the species composition among sites (Lange-Bertalot et al., 2003). In addition, diatom assemblages can also vary considerably in different geographic regions in relation to the dispersal mechanisms of species and variations of climate-related factors (such as solar radiation, cloudiness, temperature, and seasonality) (Owen, Renaut & Jones, 2008; Vanormelingen, Verleyen & Vyverman, 2008). In our opinion, these factors, although not considered in this study, could have a role in the differences found between the two geographic regions, since Auvergne is influenced by Oceanic, Continental and Mediterranean climates, and Sardinia has a Mediterranean climate.

### Relationships of diatoms with environmental variables

Among the environmental variables measured and analysed in this study, pH, conductivity and HCO_3_^−^ explained the significant amount of variance in diatom assemblages according to CCA analysis. These variables, especially pH and conductivity, are reported as significant for diatom assemblages in several geothermal systems (e.g., Owen et al., 2004; Mpawenayo, Cocquyt & Nindorera, 2005; Owen, Renaut & Jones, 2008; Angel et al., 2018; Pumas, Pruetiworanan & Peerapornpisal, 2018). In addition, total ion concentration plays a very important part in the distribution of diatoms, according to several authors (Cantonati, Gerecke & Bertuzzi, 2006; Soininen, 2007; Żelazna Wieczorek, 2011; Wojtal & Sobczyk, 2012; Wojtal, 2013). The clusters of species in the CCA plot reflected differences in the physical and chemical characteristics of the studied springs consistent with the PCA analysis based on environmental variables.

## Conclusions

The studied springs showed high species richness, especially in Sardinia, even though winter is not the most favourable season for growth and diversity of diatom flora. Significant differences were found among diatom assemblages both within each geographic region and between Auvergne and Sardinia, according to our initial hypothesis. The differences observed among sites within each region, probably due to the combined influence of several local factors, did not allow us to establish characteristic combinations of species for these environments, similar to other studies. In addition, several taxa have broad ranges of tolerance to major environmental variables and their presence is not strictly linked to thermo-mineral systems. However, some taxa can be considered more representative for springs of each region based on their greater abundance and common occurrence in multiple sites: (1) *Crenotia thermalis* for Auvergne (2) *Lemnicola exigua*, *Nitzschia amphibia*, *Nitzschia inconspicua* and *Rhopalodia operculata* for Sardinia. The level of dissimilarity found between the two geographic regions, which was unexpectedly very high, suggests that even climatic factors could be an important driver for species distribution. Among the environmental variables tested, pH, conductivity and HCO_3_^−^ were the most significant ones affecting species distribution. The present work provides a first framework on diversity, ecology and distribution of diatoms in thermo-mineral springs of different geothermal settings in Auvergne and Sardinia. The results obtained also underline the high heterogeneity of these spring environments from a biological point of view. Further studies focusing on taxonomic aspects could allow to define the identity of some abundant and dominant taxa not identified at the species level in this study. Their identification is a crucial step for a more precise ecological characterization and comparison of these peculiar spring systems.

## Additional Information and Declarations

### Data Availability

The following information was supplied regarding data availability: The raw data consist of diatom counts and results of environmental variables deposited at CNRS, GEOLAB, University Clermont Auvergne and at Laboratory of Aquatic Ecology, Department of Architecture, Design and Urban Planning, University of Sassari. Diatom count data and environmental data can also be found in Tables 2 and 3.
